# Realizing multi-functional all-optical data processing on nanoscale SiC waveguides

**DOI:** 10.1038/s41598-018-33073-y

**Published:** 2018-10-05

**Authors:** Shih-Chang Syu, Chih-Hsien Cheng, Huai-Yung Wang, Yu-Chieh Chi, Chih-I Wu, Gong-Ru Lin

**Affiliations:** Graduate Institute of Photonics and Optoelectronics, and Department of Electrical Engineering, National Taiwan University (NTU), No. 1, Sec. 4, Roosevelt Road, Taipei, 10617 Taiwan Republic of China

## Abstract

All-optical logics are realized on nanoscale SiC waveguides with add-drop micro-ring functionality, including the TE/TM polarized data decoding, the dual-port Kerr switching and gating beyond 12 Gbit/s. With employing the C-C bond enriched SiC thin film upon thermal oxide, the nonlinear refractive index of up to 2.44 × 10^−12^ cm^2^/W enables the asymmetric waveguide with polarization distinguishable transmission, which provides a polarization-selectivity to discreminate the TE/TM polarized data decoding with an nearly 9-dB extinction ratio. The TE/TM polarized decoding performance is comparable with a state-of-the-art fiberized in-line polarizer. The complementary transmission in the bus waveguide port facilitates the dual-port Kerr switching for data format conversion/inversion in both add/drop channels. Owing to the TE/TM polarization discriminated throughput, the asymmetric add-drop waveguide micro-ring also permits all-optical AND logic gating functions, where the ON-state outputs only if the pump bit is set at ON state and the probe bit with matched polarization. These results reveal the multi-functionality of the nanoscale SiC add-drop micro-ring waveguide for future photonic logics on chip.

## Introduction

In past few years, the photonic gating functionality has received more attention to meet urgent requirement of high-speed all-optical data transmission and processing on chip in the coming era^1^. Numerous literatures related to the research on silicon photonic integrated circuits also thrives owing to its superior compatibility with currently available CMOS fabrication technology^2–4^. Among all demonstrated device schemes, the ultrafast optical switches and modulators have attracted more interests^1^. To realize large-scale photonic integrated circuits on chip, the polarization-handling is an important issue as most of the optoelectronic components are polarization-sensitive^5^. Typically, the on-chip polarizer can be made by a planar waveguide with high-aspect-ratio^6–8^; however, these waveguide structures exhibit high birefringence and different confinements for TE and TM modes so as to result in different propagation and bending losses. For such a high-aspect-ratio waveguide based polarizer, the coupling of light into the flat planar waveguide becomes more difficult than that into the channel waveguide. Alternatively, splitting the TE/TM-mode was implemented with asymmetrical coupling between a hybrid plasmonic waveguide and a strip dielectric waveguide^9^. Due to the high birefringence of the hybrid plasmonic waveguide, only the TM-mode dependent light in a strip dielectric waveguide can achieve the phase matching in the hybrid plasmonic waveguide. Previously, Almeida *et al*. demonstrate different switching mechanisms in Si waveguide by launching changing the pumping power at ~1 GW/cm^2^ or lower to induce Kerr effect for phase shift up to 180°, whereas the FCA effect is induced to decay the modulation speed down to 1 GHz by enlarging the pumping power above 30 GW/cm^2 ^^10^. The on-off extinction ratio can be achieved to 13 dB with using the phase modulation to implement the Si waveguide Mach-Zehnder interferometer^10^. Concurrently, Almeida *et al*. achieved the all-optical switching in Si waveguide resonator by intensively pumping the waveguide to cause a refractive index change of 4.8 × 10^−4^ with associated modulation depth enhancing up to 94%^11^. In addition, Lin *et al*. performed the SiO_2_/SiO_x_/SiO_2_ strip-loaded waveguide to demonstrate optical amplification with a small-signal power gain as high as 9.5 dB^12^. Huang *et al*. observed the FCA effect in SiC micro-ring for implementing the all-optical data switching, which provides a signal-to-noise ratio (SNR) can be achieved as high ae 5 dB under the 1-Gbit/s PRZ-OOK data transmission^13^. In addition, the polarization modulation can also be applied with versatile approaches. Huang *et al*. used a multi-mode interfered waveguide coupler on silicon-on-insulator (SOI) to achieve highly polarized extinction ratios of 20 dB for TE-mode and 15 dB for TM-mode^14^. Xu *et al*. applied the asymmetrical directional couplers with bending and tapering waveguide to demonstrate a TE-selective polarizer with 30-dB extinction by detuning the phase mismatch between TE/TM polarizations^15^. In addition, Xie *et al*. employed the four-wave mixing effect to demonstrate the polarization-multiplexed non-return-to-zero amplitude-shift keying with a data rate of 20 Gbit/s^16^. On the other hand, Kerr effect is another method to perform all-optical switching. Ikeda *et al*. employed the SiN_x_ add-drop micro-ring with a nonlinear refractive index of 2.4 × 10^−15^ cm^2^/W to demonstrate on-off-keying (OOK) data transmission at 1 Gbit/s^17^. Huang *et al*. increased the graphite-like C-C bonds in C-rich SiC_x_ to enhance its nonlinear refractive index up to 6.6 × 10^−13^ cm^2^/W for performing the pulsed return-to-zero OOK (PRZ-OOK) data transmission at 12 Gbit/s^18^. Moreover, all-optical wavelength and format converted functionalities have also emerged to develop silicon photonic integrated circuits. Dekker *et al*. utilized the 1.55-μm femtosecond pulse to induce the Kerr effect in SOI waveguides for the wavelength up- and down-conversion^19^. Xu *et al*. employed the free-carrier dispersion in Si micro-ring waveguide to perform all-optical wavelength converter, and the modulated data rate can be achieved to 0.9 Gbit/s under a CW control power of 4.5 mW^20^. Astar *et al*. employed both cross-phase modulation and four-wave mixing to perform all-optical data conversion from non-return-to-zero OOK (NRZ-OOK) to RZ-OOK with a receiving sensitivity gain of 2.5 dB^21^. In addition, Huang *et al*. also employed the FCA effect in SiGeC waveguide to achieve the PRZ-OOK data format inversion up to 6 Gbit/s^22^. The all-optical logic gate is necessary to play a key role for ultrafast data processing in optical quantum computing and communication, which was mainly implemented with semiconductor optical amplifier (SOA) or periodically poled lithium niobate (PPLN) devices^23,24^. In particular, the SOA-based all-optical logic gates has achieved multi-functional OR, NOT and logics by utilizing cross-phase modulation (XPM), cross-gain modulation (XGM) and four-wave-mixing (FWM) effects^23^, and the PPLN-based logics including AND and NAND functions have also been demonstrated lately^24^. More recently, silicon waveguide based logics have also been demonstrated by utilizing versatile device structures including cascaded micro-rings or Mach-Zehnder interferometers^25^. Among these logic devices, different optical nonlinear effects such as FWM, two photon absorption (TPA) and plasmas dispersion based logics have been employed to demonstrate faster operation speed than electro-optic effect^25–29^. Nevertheless, the Kerr switching logic based on the add-drop micro-ring structure was seldom discussed for potential application in high-speed operation.

Unfortunately, using bulk silicon substrate for device fabrication has caused some drawbacks to limit its application in high-speed data processing. Remarkably, the bulk silicon inherently exhibits long minority carrier lifetime ranged from µs to ms, which restricts the operation bandwidth to only few MHz. Many researches were proposed to release such a bandwidth limitation by either increasing the drift velocity or shortening the carrier lifetime^30–33^. Among different mechanisms, the Si-QD embedded optoelectronic devices with strong quantum confinement effect have the most potential for signal processing beyond GHz. However, the bulk silicon also exhibits the two-photon absorption induced free-carrier absorption (FCA) effect^34–36^, which inevitably results in a recombination (or dissipation) time of ~1 ns to further limit the modulation speed. To meet this demand, nonlinear Kerr switching is proposed as an alternative to the FCA switching for implementing the ultrafast all-optical modulation^3,37^. Nevertheless, the nonlinear refractive index of bulk silicon is only 4 × 10^−14^ cm^2^/W at 1550 nm^3^, which is insufficient to realize significant nonlinear optical switching. Recently, the carbon-rich silicon carbide (C-C bond enriched SiC) with abundant graphene-like C-C sp^2^ bonds to provide strong optical nonlinearity was proposed^38^.

In this work, an asymmetric C-C bond enriched SiC add-drop micro-ring is demonstrated to implement TE/TM polarized data decoding, wavelength conversion, format inversion and all-optical logic operations. To perform the TE/TM polarized decoding, the polarization-selective property of the TE- and TM-mode related transmission spectra is utilized to attenuate a specific TE- or TM-mode intensity for polarization selective data receiving. The add-drop micro-ring can serve as an input polarization-angle dependent TE/TM switchable decoder. The add-drop micro-ring related TE/TM polarized decoding performance at data rates of 4, 6 and 12 Gbit/s is comparable with a commercial in-line polarizer. The C-C bond enriched SiC add-drop micro-ring based Kerr switch also enables the wavelength conversion at date rates of 1.2, 6 and 12 Gbit/s, owing to its transient nonlinear refractive index change with incoming data. Benefiting from the complementary transmission spectra at bus and micro-ring waveguide ports, and the Kerr switching effect enables the simultaneous TE/TM mode selection and data format inversion in the C-C bond enriched SiC micro-ring port. Only when the input probe polarization matches the mode dependent spectral dip, the probe can be cross-wavelength switched by pump. By controlling the polarization and the on/off status of pump and probe, the output of the asymmetric add-drop micro-ring waveguide would implement the functionality of all-optical AND logic gate. Such a C-C bond enriched SiC add-drop micro-ring can thus serve as a multi-function at data processor for photonic integrated circuit to be developed in next generation.

## Results

### Characteristic Analysis and Structural Design of C-C bond enriched SiC Based Add-drop Micro-ring

**Figure 1:**
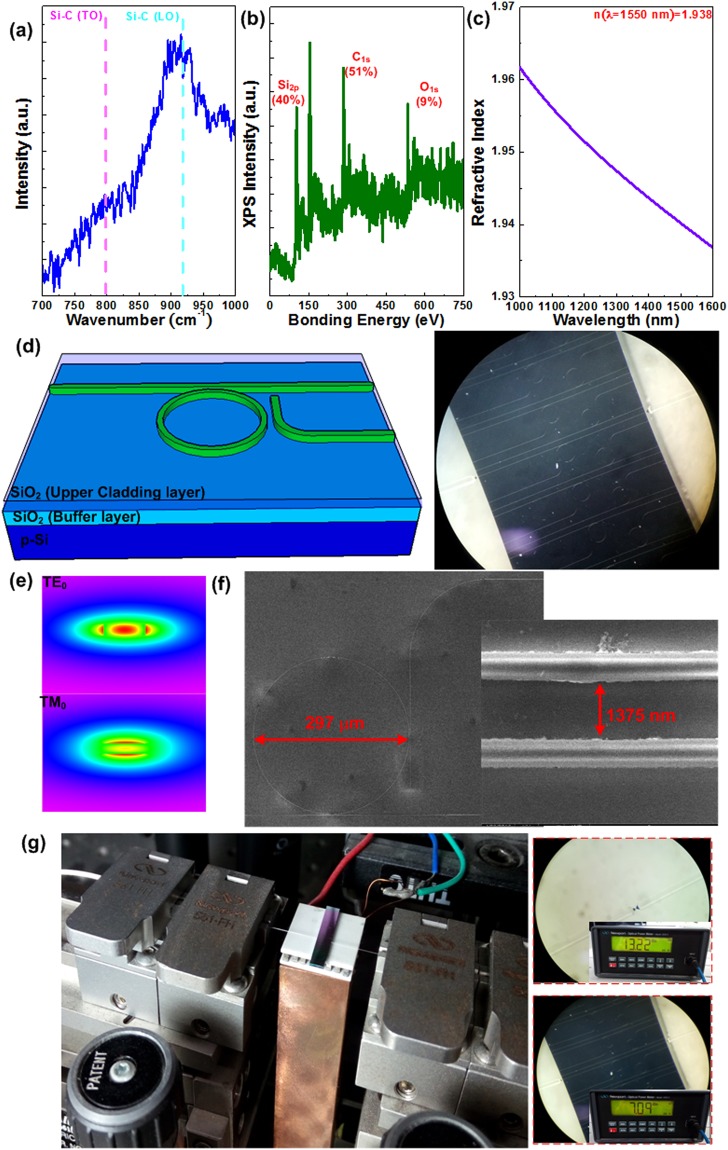
Material characteristics of the C-C bond enriched SiC film and design of the add-drop micro-ring. (**a**) The Raman spectrum of the SiC film. (**b**) The XPS spectrum of the SiC film. (**c**) The refractive index spectrum of the SiC film. (**d**) The schematic diagram and photograph of the SiC add-drop micro-ring. (**e**) The simulation profiles for TE_0_ and TM_0_ modes. (**f**) The SEM image of designed add-drop micro-ring. (**g**) The add-drop micro-ring on the lensed-fiber alignment system.

First of all, the Raman scattering spectroscopy of a 350-nm SiC film deposited on a thin quartz plate was performed as shown in Fig. 1(a). Two peaks at around 795 and 920 cm^−1^ are observed, which respectively corresponds to the transverse optical (TO) and the longitudinal optical (LO) modes of Si-C bonds. These measured Raman peaks are broadened because of nearly amorphous structure of the as-grown SiC film. Later on, the X-ray photoelectron spectroscopy (XPS) was performed to analyze the composition of the SiC film, which provides Si_2p_ and C_1s_ orbital electron related peaks at 99.8 and 281.9 eV, indicating that the atomic ratios of Si and C are 40% and 51%, respectively, as shown in Fig. 1(b). More C atoms than Si atoms exist in such a C-C bond enriched SiC film, which form more graphene-like C-C bonds to enhance its optical nonlinearity. The dispersion can be observed from the wavelength dependent refractive index spectrum of the C-C bond enriched SiC film shown in Fig. 1(c), which reveals a refractive index of 1.938 at a wavelength of 1550 nm. The geometric structure of designed bus/ring waveguide with height of 350 nm and width of 500 nm is shown in Fig. 1(d), in which the diameter of ring resonator is 300 µm and the gap spacing between bus/ring waveguide (or ring/L-shaped waveguide) is 1400 nm. To increase the coupling efficiency, an inversed taper with its width increasing from 200 to 500 nm was utilized. The right part of Fig. 1(d) shows the surface images of SiC add-drop micro-ring. The RSoft commercial tool with a simulation based on beam propagation method was employed to guarantee the single-mode operation of the design. Figure 1(e) also shows the simulated field profiles for TE_0_ and TM_0_ modes. Due to the asymmetric waveguide structure, the designed waveguide prefers the TE_0_ mode to exhibit better modal confinement than the TM_0_ mode. Figure 1(f) shows the SEM image of the designed add-drop micro ring. After measurement, the ring diameter, the waveguide width and gap spacing are determined as 297 µm, 562 nm and 1375 nm, respectively. Figure 1(g) is the photograph illustrating the add-drop micro-ring aligned with the lensed fibers firmed on translation stages. The total insertion loss is about 6.13 dB, after measuring the coupling loss induced by lensed fibers, and the propagation loss of the add-drop micro-ring. The cut-back analysis indicates that the propagation loss is only 0.43 dB/mm in addition to the lensed-fiber-to-waveguide coupling loss of ~3 dB/facet^39^. Therefore, the propagation loss in C-C bond enriched SiC based waveguide can be ignored.

### TE- and TM-mode related Transmission Spectra of the Add-drop Micro-ring

**Figure 2:**
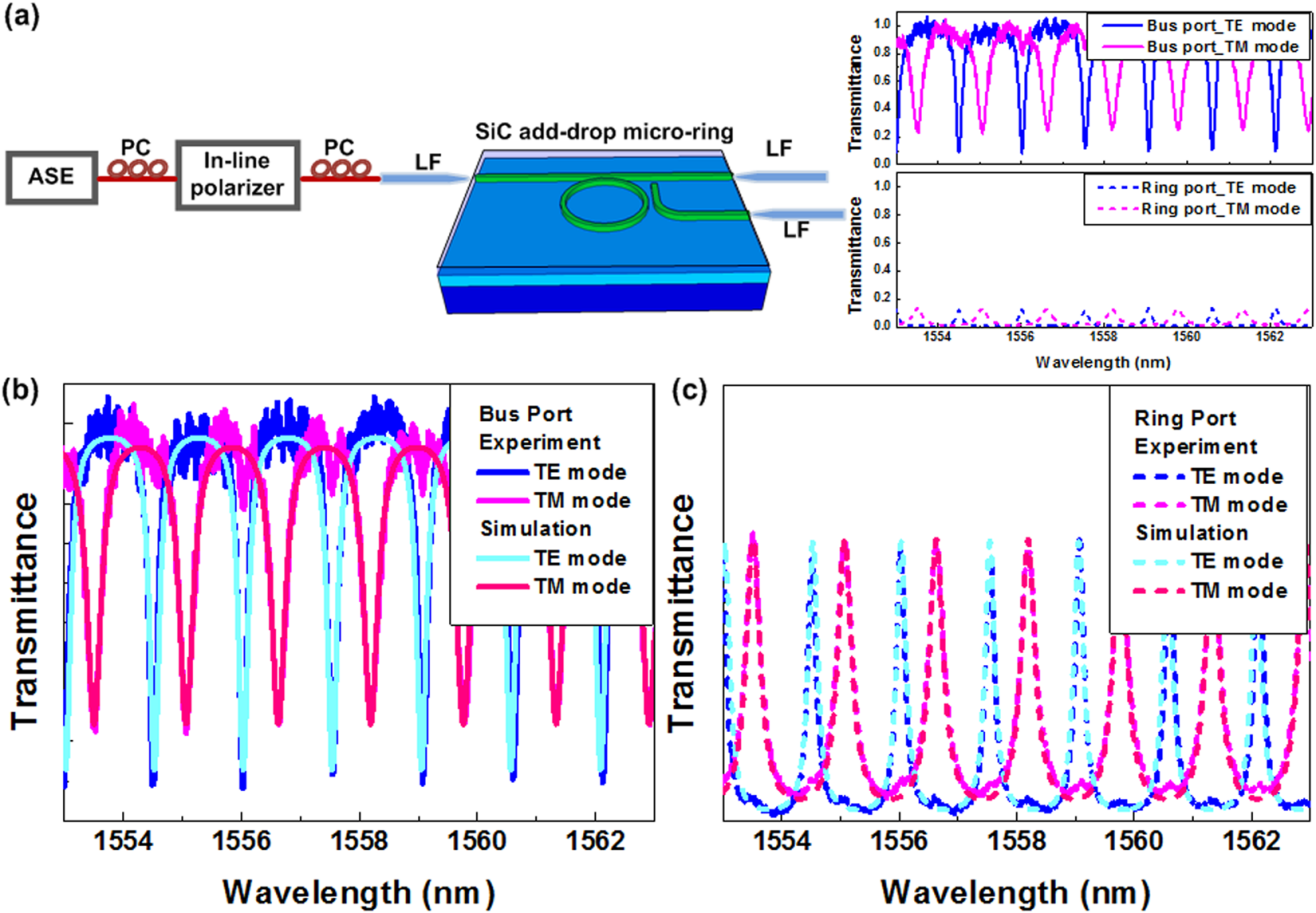
The measured and fitted transmission spectra of the C-C bond enriched SiC based add-drop micro-ring. (**a**) The experimental setup for measuring the TM/TM-mode dependent transmission spectrum and The measured TE/TM-mode dependent transmission spectrum. The fitted transmission spectra for TE and TM modes at (**b**) bus and (**c**) ring ports.

The experimental setup for measuring the TE- and TM-mode related transmission spectra caused by the add-drop micro-ring is shown in Fig. 2(a). Initially, the throughput of incoming ASE signal passes through an in-line polarizer to form a unipolarized light, and a pure TE- (or TM-mode) light was obtained after properly tuning the polarization status, which launches into the SiC based add-drop micro-ring resonator for measuring the transmission spectrum, as shown in Fig. 2(a). The notched dips observed from the output port of the bus waveguide result from the light at specific wavelengths trapped in the micro-ring resonator. The trapped light can be partially coupled out from the micro-ring resonator, and two independent transmission spectra for TE- and TM-mode were observed during the input polarization adjustment. In principle, the normalized transmission spectra obtained from bus and ring ports of an add-drop micro-ring can be simulated by the following equations^3,40,41^,

1 $${T}_{bus}=\frac{{r}_{2}^{2}{a}^{2}-2{r}_{1}{r}_{2}a\,\cos (\varphi +{\varphi }_{{\rm{0}}})+{r}_{1}^{2}}{1-2{r}_{1}{r}_{2}a\,\cos (\varphi +{\varphi }_{{\rm{0}}})+{({r}_{1}{r}_{2}a)}^{2}},$$

2 $${T}_{ring}=\frac{(1-{r}_{1}^{2})(1-{r}_{2}^{2})a}{1-2{r}_{1}{r}_{2}a\,\cos (\varphi +{\varphi }_{{\rm{0}}})+{({r}_{1}{r}_{2}a)}^{2}},$$

In Eqs (1) and (2), *T*_*bus*_ and *T*_*ring*_ respectively denote the transmittances of bus and ring ports, *r*_1_ (or *r*_2_) the self-coupling coefficient in the bus port (or ring port), *k*_1_ (or *k*_2_) the cross-coupling coefficient with the relationship between self- and cross-coupling coefficients of *r*_1_^2^ + *k*_1_^2^ = 1 (or *r*_2_^2^ + *k*_2_^2^ = 1) when ignoring the coupling loss. In addition, *ϕ* is the run-trip phase shift correlated to group index *N*_*g*_ by *ϕ* = *βL* = 2π*N*_*g*_*L*/*λ*, where *β* denotes the propagation constant, *L* = 2π*R* the cavity length with *R* representing the radius of the resonator and *λ* the input wavelength. *ϕ*_0_ is a random parameter denoting the initial phase shift, *α* is the amplitude attenuation expressed as *α*^2^ = exp(−*αL*) in the resonator with an attenuation coefficient of *α*. For convenience, the detailed parameters of the SiC based add-drop micro-ring resonator for simulation are listed in Table 1. Typically, the asymmetric waveguide induces different TE- and TM-mode group indices in the micro-ring to split their transmission comb spectra. If the waveguide design can be precisely detuned to achieve the symmetric structure, the transmission comb can ideally overlap with each other in piratical case.

**Table 1:** The simulated parameters of the C-C bond enriched SiC based add-drop micro-ring resonator.

SiC	TE mode	TM mode
R, Radius of Micro-ring (μm)	150	
α, Amplitude attenuation (μm^−1^)	5 × 10^−4^	7.5 × 10^−4^
N_g_, Group index	1.705	1.645
Δλ, FSR (nm)	1.5048	1.5596
δλ, FWHM (nm)	0.1727	0.3016
y_bus_ = cos(*κ*_1_*l*)	0.8779	0.8664
y_ring_ = cos(*κ*_2_*l*)	0.9595	0.9272
Q, Quality Factor	7919	5398
Magnification factor, M	1.1781	0.5565
ER_bus_ (dB)	8.76	5.86

As a result, the transmission spectra contributed by TE and TM modes can be simulated by using different group indices with *N*_*g*_^*TE*^ = 1.705 and *N*_*g*_^*TM*^ = 1.645, respectively, as also shown in Fig. 2(b,c). Note that the SiC based add-drop micro-ring resonator favors the TE_0_ mode more than the TM_0_ mode because of its asymmetric waveguide structure, which indicates a FWHM of 0.1727 nm, a Q-factor of 7919 and the modal extinction ratios (MERs) of 8.76 dB and 0.6 dB for the TE_0_ mode in bus and ring ports, respectively. In contrast, the TM_0_ mode incidence only shows Q-factor of 5398 and MER of 5.86 and 0.56 dB for the bus and ring ports, respectively. Note that a micro-ring resonator with extremely high Q-factor is unsuitable for performing such kind of wavelength-converted data processing at high switching speed. The relationship between the Q-factor and switching speed has been discussed in previous work. The photon lifetime (τ_p_) in a micro-ring resonator can be described^4^.

3 $${\tau }_{p}\approx \frac{Q{\lambda }_{0}}{2\pi c},$$

where Q denotes the quality factor of the SiC based micro-ring add-drop, *λ*_0_ the wavelength, c the light speed. From the abovementioned formula, the photon lifetime is increased with enlarging the Q-factor of SiC based micro-ring add-drop. Typically, the modulation speed is inversely proportional to the photon lifetime in the SiC, as the data delivered by circulated photons keeps superimposed each other to induce the data waveform distortion for error bit receiving. Therefore, the higher Q factor for the SiC based add-drop micro-ring add-drop is unsuitable for performing the high-speed all-optical data switching.

### TE- and TM-mode related Transmission Spectra of the Add-drop Micro-ring

**Figure 3:**
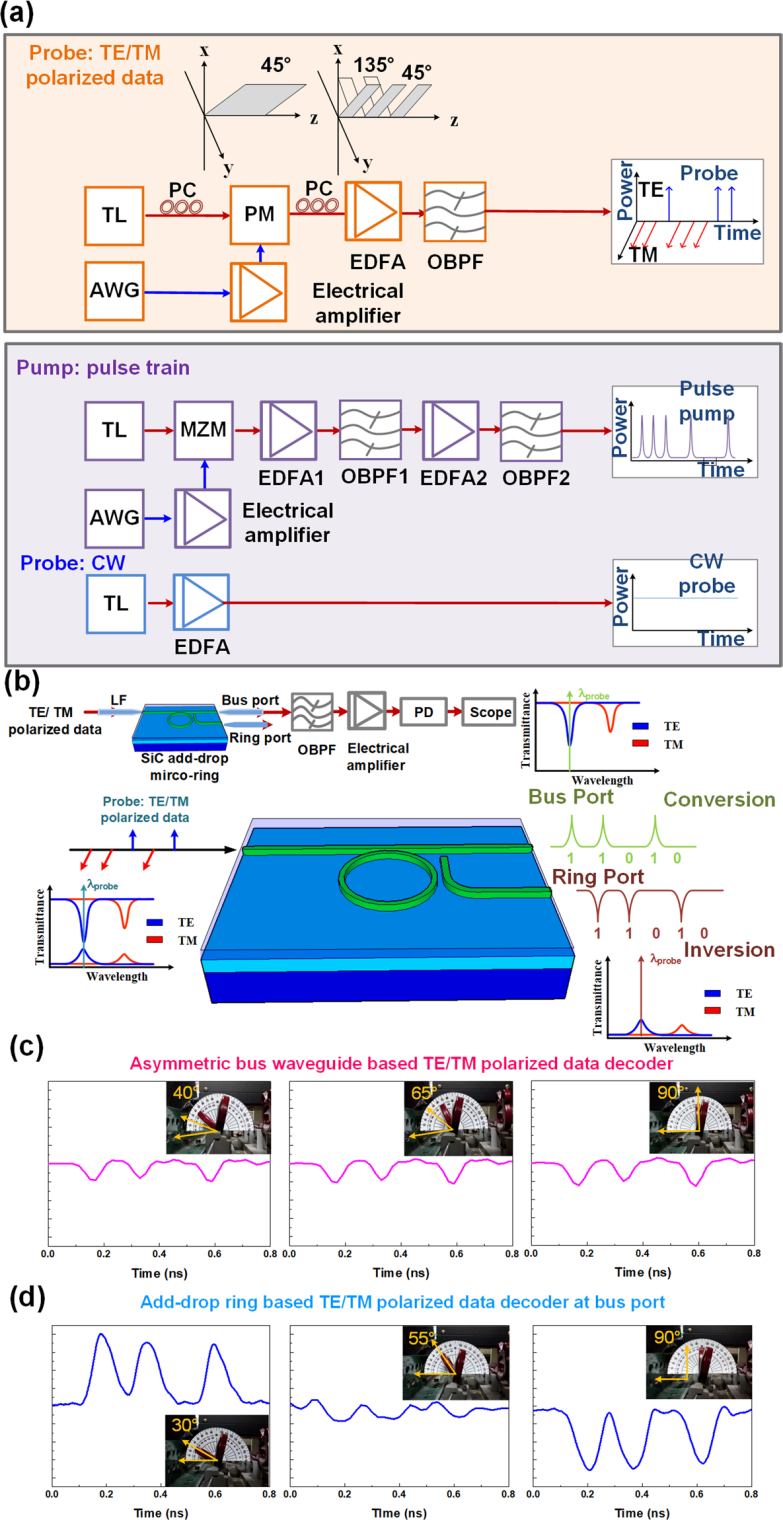
The schematic diagram of add-drop micro-ring based all-optical TE/TM polarized decoder, all-optical Kerr switch and all-optical logic application. (**a**) The schematic diagram is mainly divided into three parts; Upper: the generation of optical TE/TM polarized data. Middle: a pumping light carries a highly peak power pulse-train. Lower: a CW probe source. (**b**) Schematic diagram for polarization-to-intensity switching with the proposed polarization-selective add-drop micro-ring. The 12-Gbit/s TE/TM polarized data is decoded by (**c**) an asymmetric bus waveguide and (**d**) add-drop micro-ring based TE/TM polarized decoder at different polarization angles of input light.

The two sets of TE- and TM-mode related transmission spectra in the add-drop micro-ring are separated with each other due to their different group refractive indices and deviated free spectral ranges (FSRs). Therefore, the add-drop micro-ring provides two distinct groups of transmission spectral combs for enabling the TE/TM polarized data decoding. To demonstrate, a CW light with 45°-incident angle is encoded by a phase modulator to form the encoded data stream for optical TE/TM polarized data generation, as shown in the upper row of Fig. 3(a).

Then, the optical TE/TM polarized data stream is sent into the add-drop micro-ring, as shown in Fig. 3(b). If the wavelength of encoded data stream coincides with one of the TE-mode related notched dips, only the TM-mode component can pass through the add-drop micro-ring and the TE-mode dependent light would be weakened. This leads to the decoding from the TE/TM polarized data to the on-off keying (OOK) data. Moreover, the throughput data format observed in the bus port is reversed with that in the ring port because they have complementary transmission spectra. That is, the add-drop micro-ring concurrently enables all-optical wavelength conversion and format inversion of data stream in bus and ring ports, respectively. Particularly, the asymmetric add-drop micro-ring also serves as a TE/TM switchable decoder with inserting and tuning a polarization controller, whether the format of the output signal can be preserved or inverted on the basis of the input polarization relative to the waveguide preference.

When operating the 12-Gbit/s TE/TM polarized decoding, the results of a typical asymmetric bus waveguide and the proposed add-drop micro-ring with a specific data stream of “01010010” at different input polarization angles are compared in Figs 3(c) and 3(d). Note that the decoding performance of the bus waveguide is almost independent from the polarization angle turning as the asymmetric waveguide induces different propagation losses for TE and TM modes. On the contrary, the add-drop micro-ring exhibits polarization sensitive behavior on TE/TM polarized decoding. When adjusting the wavelength at TE-mode notched dip and properly detuning the polarization angle to 30°, the converted data with maximal amplitude is observed from the add-drop micro-ring, as shown in the left part of Fig. 3(d). Increasing the input polarization angle weakens the decoded data amplitude because of the same TE and TM components. Continuously tuning the polarization angle to 90°, which becomes perpendicular with the preferred one in the add-drop micro-ring, inverts the format of decoded data with the maximal value, as shown in the right of Fig. 3(d). Such a polarization-selective add-drop micro-ring serves as the TE/TM switchable decoder to provide an OOK data output with an extinction ratio (ER) of 6.67 dB and an amplitude of 200 mV higher than those of 4.26 dB and 60 mV directly decoded from the bus waveguide.

**Figure 4:**
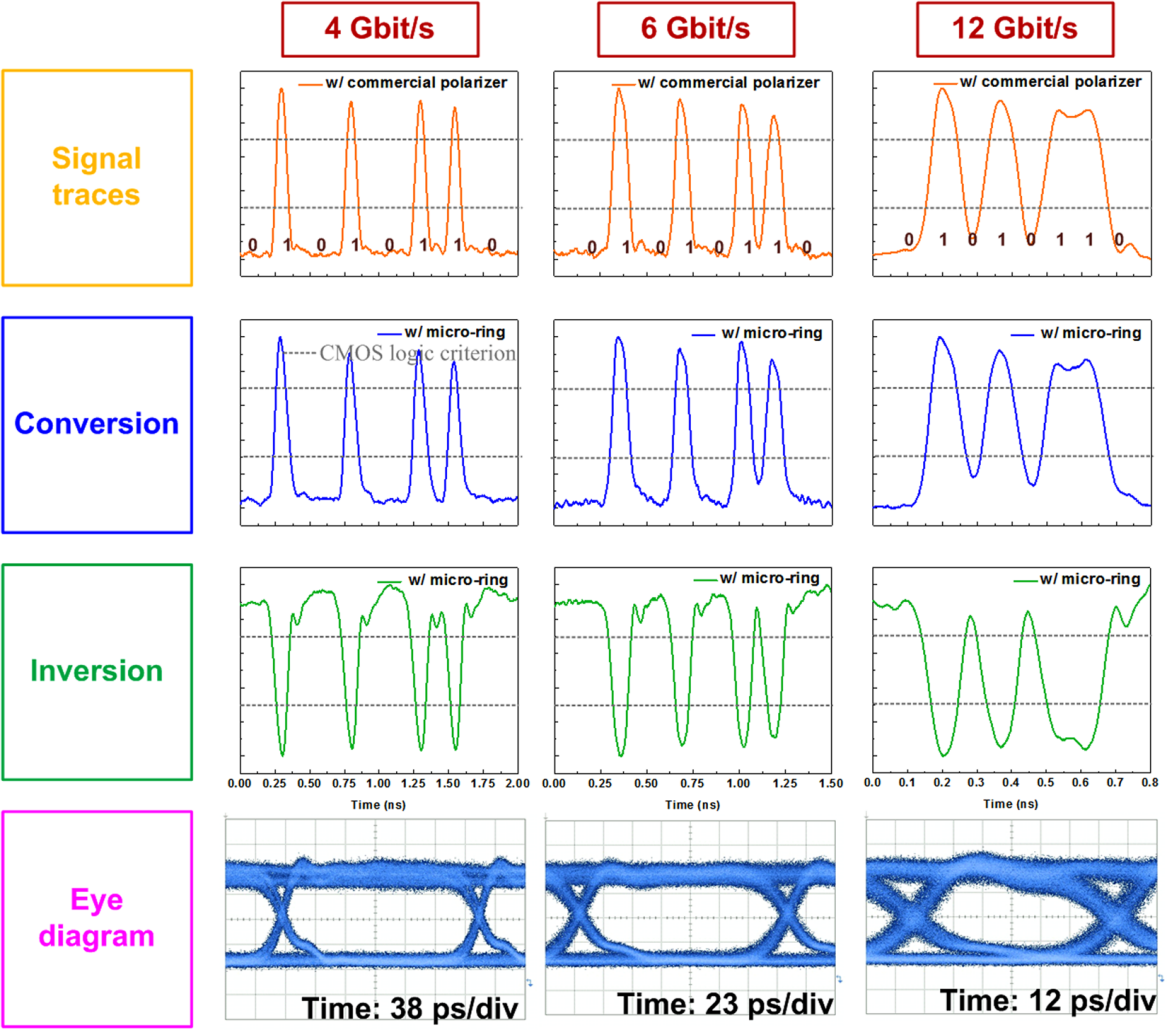
The TE/TM polarized decoding performance of the add-drop micro-ring. A TE/TM polarized data with a specific data stream of “01010110” at different data rates (4, 6 and 12 Gbit/s) is decoded by a commercial 25-dB in-line polarizer (first column) and the add-drop micro-ring (2^nd^ and 3^rd^ column). The decoded converted and inverted signal by the add-drop micro-ring are shown in the 2^nd^ and 3^rd^ column of the figure. The diagrams in the last column of the figure show the stacked traces of 4, 6 and 12-Gbit/s TE/TM polarized data stream with a pattern length of 2^7^-1 decoded by the add-drop micro-ring.

Moreover, the TE/TM polarized decoding performances of the add-drop micro-ring at different data rates (4, 6 and 12 Gbit/s) are shown in Fig. 4. The first row of Fig. 4 shows the incoming TE/TM polarized signal with a specific data stream of “01010110” and a pulsewidth of 83 ps after decoded by a commercial 25-dB in-line polarizer. For comparison, the traces of format preserved and inverted data streams after TE/TM polarized decoding with the add-drop micro-ring are shown in the second and the third rows of Fig. 4, respectively. As the data rate of the input data stream increases from 4 to 12 Gbit/s, both of the commercial in-line polarizer and the add-drop micro-ring can successfully decode the TE/TM polarized data with standard CMOS logic qualified data criterion^42^. By lengthening the pattern length of the probe TE/TM polarized signal to 2^7^-1, the eye diagrams of add-drop micro-ring converted 4-, 6, and 12-Gbit/s data are shown in the fourth rows of Fig. 4, and the related parameters are summarized in Table 2. These eye diagrams exhibits similar ER of ~7.5 dB, which are smaller than the MER maximum of 8.76 dB obtained at TE-mode dip because of the existence of DC offset. Note that the RMS jitter of the converted 4, 6, and 12-Gbit/s data are 9.0, 11.8 and 7.3 ps respectively.

**Table 2:** The related parameters of the eye diagram for 4, 6 and 12-Gbit/s TE/TM polarized data decoded by the add-drop micro-ring.

	4 Gbit/s	6 Gbit/s	12 Gbit/s
ER (dB)	7.51	7.42	7.36
SNR	9.03	8.51	7.28
Jitter RMS (ps)	3.25	2.97	3.45
Rising time (ps)	38	34.5	28.5
Falling time (ps)	57	54.6	39
Cross point	52%	52%	46%
BER	5.19 × 10^−9^	1.55 × 10^−7^	5.32 × 10^−5^

### Cross-wavelength 10-Gbit/s Data Conversion and Inversion with Kerr Effect

**Figure 5:**
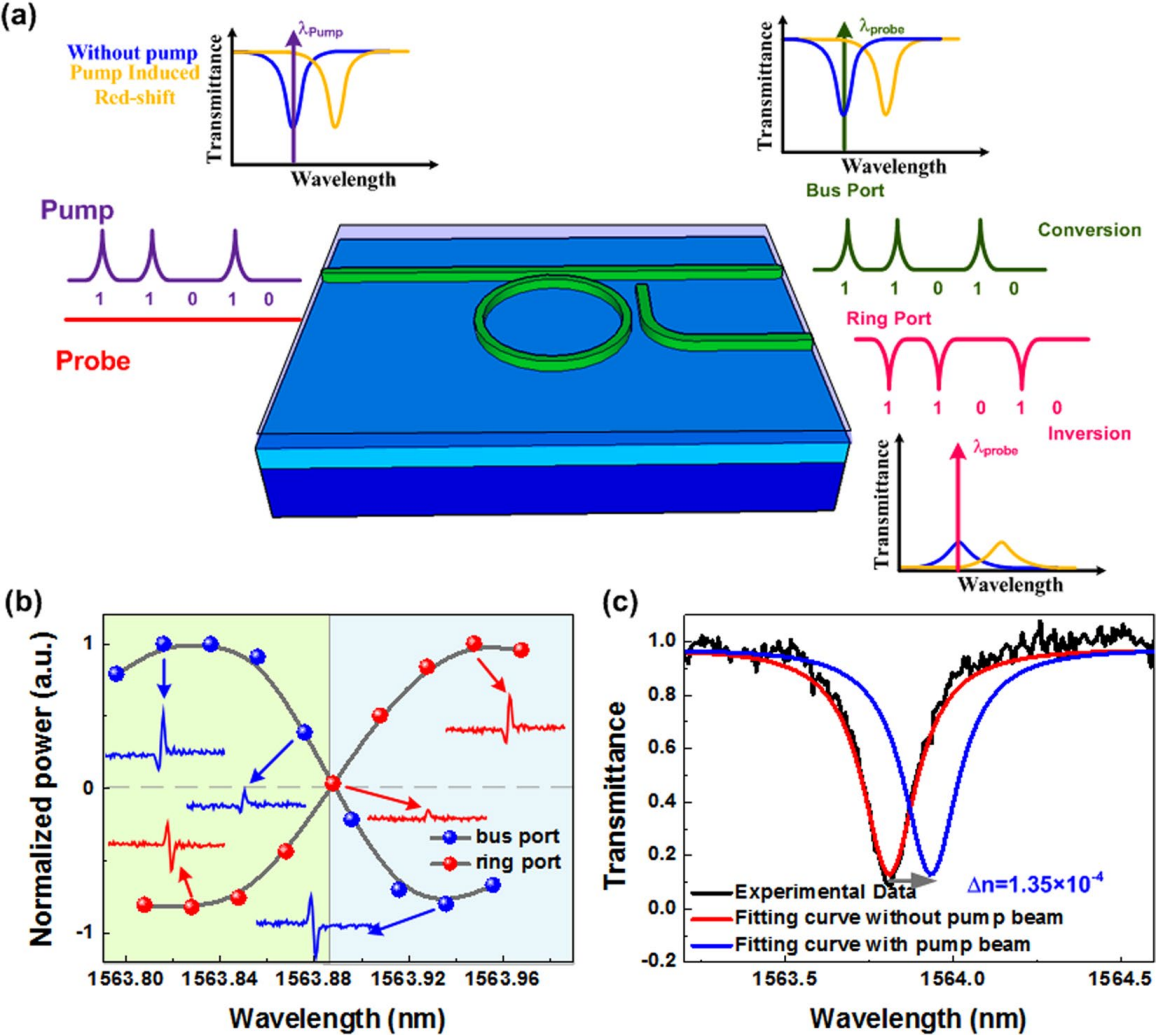
C-C bond enriched SiC add-drop micro-ring based Kerr switch. (**a**) The schematic diagram shows C-C bond enriched SiC based add-drop micro-ring for simultaneously converted and inverted data conversion with nonlinear Kerr effect. (**b**) The amplitude of modulated signals at different probe wavelengths in the bus and ring port; insert: The bus- and ring-port modulated probe traces at different probe wavelengths. (**c**) The transmission spectra of the C-C bond enriched SiC based add-drop micro-ring without and with pump beam.

The experimental setup for implementing the data switching in the C-C bond enriched SiC based add-drop micro-ring with enhanced Kerr nonlinearity is shown in Fig. 5(a). Both an intense pump and a CW probe light with properly controlled polarization via two in-line polarizers are 50/50 coupled into the polarization-sensitive add-drop micro-ring. Note that the intense pump pulse results in transient change on refractive index as well as the notch wavelength of the C-C bond enriched SiC based add-drop micro-ring, which consequently red-shifts the transmission combs so as to all-optically switch the probe. The initial wavelength of probe decides whether the data format is preserved or inverted as it sets within or out of the notched dip of the add-drop micro-ring^4^. With the bus and L-shaped dual output ports, the add-drop micro-ring simultaneously enables the optical data streams at different wavelength channels with preserved and inverted formats. To check the enhanced Kerr nonlinearity, the conversion performance in the bus and ring ports of the add-drop micro-ring was characterized by a single-bit pulse initially, as shown in Fig. 5(b). When both of the pulse and probe wavelengths are set at the same transmission notch of the add-drop micro-ring, the probe would be modulated by the pump to convert the data in the bus port and invert the data in the ring port, as shown in the insert of Fig. 5(b). By detuning the probe wavelength out of the transmission notch, the converted data format in bus and ring ports immediately exchanges each other, as plotted in Fig. 5(b).

From the wavelength spacing of 0.12 nm between format preserved maximum conversion and format inverted maximum inversion, the transmission spectral shift in the add-drop micro-ring owing to the pump induced Kerr nonlinearity change is observed, as shown in Fig. 5(c). The corresponding group refractive index change is ΔN_g_ = 1.35 × 10^−4^, and the nonlinear refractive index (n_2_) of the C-C bond enriched SiC can be calculated by using n_2_ = ΔN_g_/(M × I_peak_), in which M denotes the field magnification factor of the add-drop micro-ring and I_peak_ the pulse intensity coupled into the resonator. During experiment, M = 1.18 is obtained for the add-drop micro-ring. With the total loss of ~6.7 dB, the peak pump power of 1.078 W, and the waveguide effective area of 2.3 µm^2^, the nonlinear refractive index of the C-C bond enriched SiC is characterized as 2.44 × 10^−12^ cm^2^/W.

**Figure 6:**
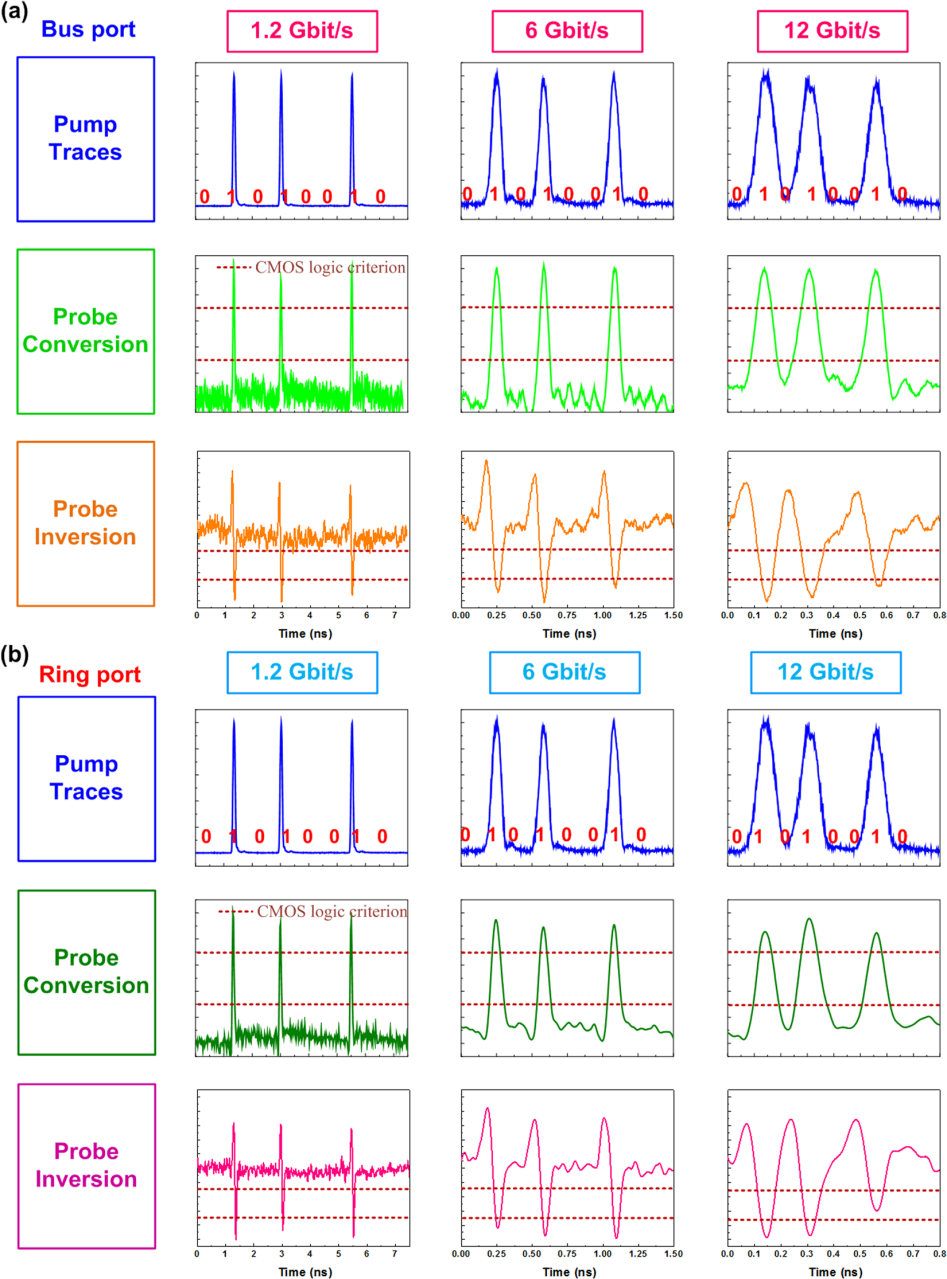
The Kerr switching performance with C-C bond enriched SiC the add-drop micro-ring. (**a**,**b**) The bus- and ring-port Kerr switching performance is shown respectively. The pulse traces of 1.2, 6 and 12-Gbit/s PRZ-OOK data are plotted in the first row of the figure. The modulated probe signals in conversion (2^nd^ row) and inversion (3^rd^ row) format are given.

**Figure 7:**
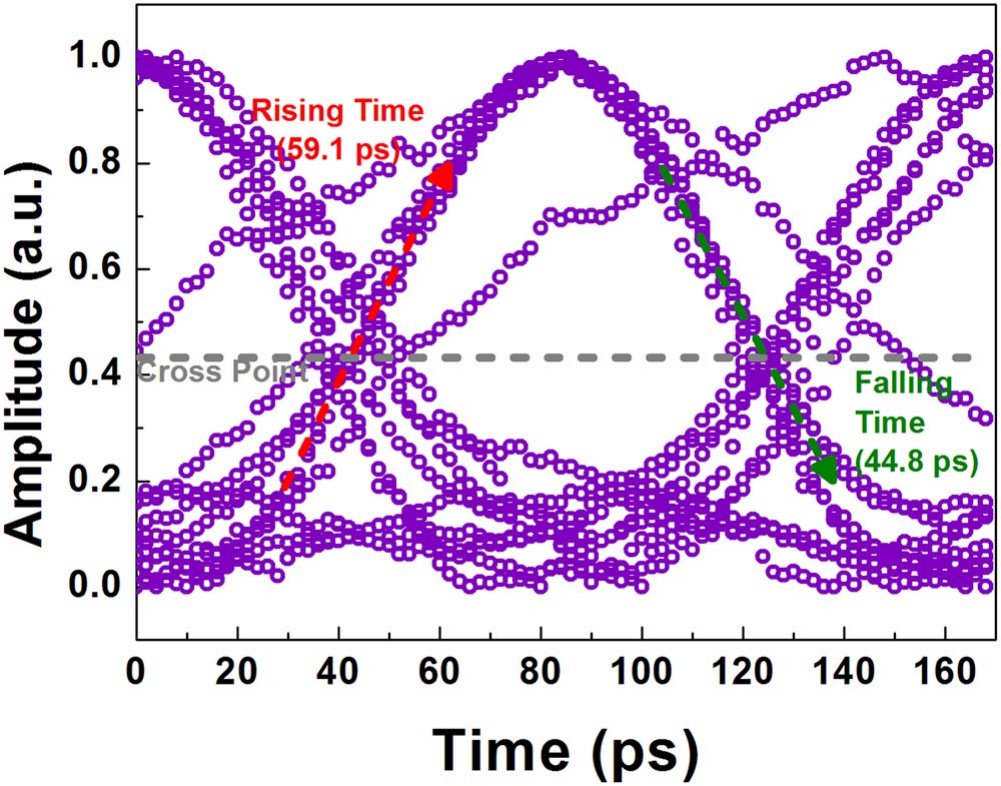
The eye diagram of 12-Gbit/s converted PRZ-OOK with C-C bond enriched SiC based add-drop micro-ring. The diagram shows the stacked traces of 12-Gbit/s PRZ-OOK converted by C-C bond enriched SiC add-drop micro-ring based Kerr switch.

Subsequently, the high-speed data switching with a specific pattern of “01010010” at different data rates of 1.2, 6 and 12 Gbit/s are analyzed, as shown in Fig. 6(a,b). Note that the initially rising part of the inverted data trace is mainly contributed to interference from closely overlap between the TE and TM related dips. When increasing the pump date rate up to 12 Gbit/s, the switched probe output becomes distorted more than ever with degrading eye patterns failed to follow up the original ones as shown in Fig. 7. As a result, the eye diagram of converted 12-Gbit/s data presents an ER of 4.48 dB, a SNR of 5.56 dB, and a cross point of 42%, and the RMS jitter, rising and falling time are 8.12 ps, 59.1 ps and 44.8 ps, respectively. This results can obtain the corresponding BER of 7.49 × 10^−5^. From Eq. (3), this SiC based add-drop micro-ring with a Q-factor of 7919 can support the data transmission with a modulation bandwidth of 153 GHz. When decreasing the Q-factor, the modulation bandwidth can be increased but the coupling ratio is also increased to reduce the modulation depth. This trade-off between the modulation bandwidth and modulation depth is considered when trying to improve the bandwidth. In addition, one of the solutions to improve the operation speed is utilizing the new data formats with higher spectral usage efficiency, including the four-level pulse amplitude modulation (PAM-4) and the quadrature amplitude modulation orthogonal frequency division multiplexing (QAM-OFDM) data formats. For example, the PAM-4 data format can increase twice the transmission capacity over the NRZ-OOK data format. Under the finite modulation bandwidth of device, the spectral usage efficiency is an important issue for the silicon photonics in the future.

### All-optical logic gate application

**Figure 8:**
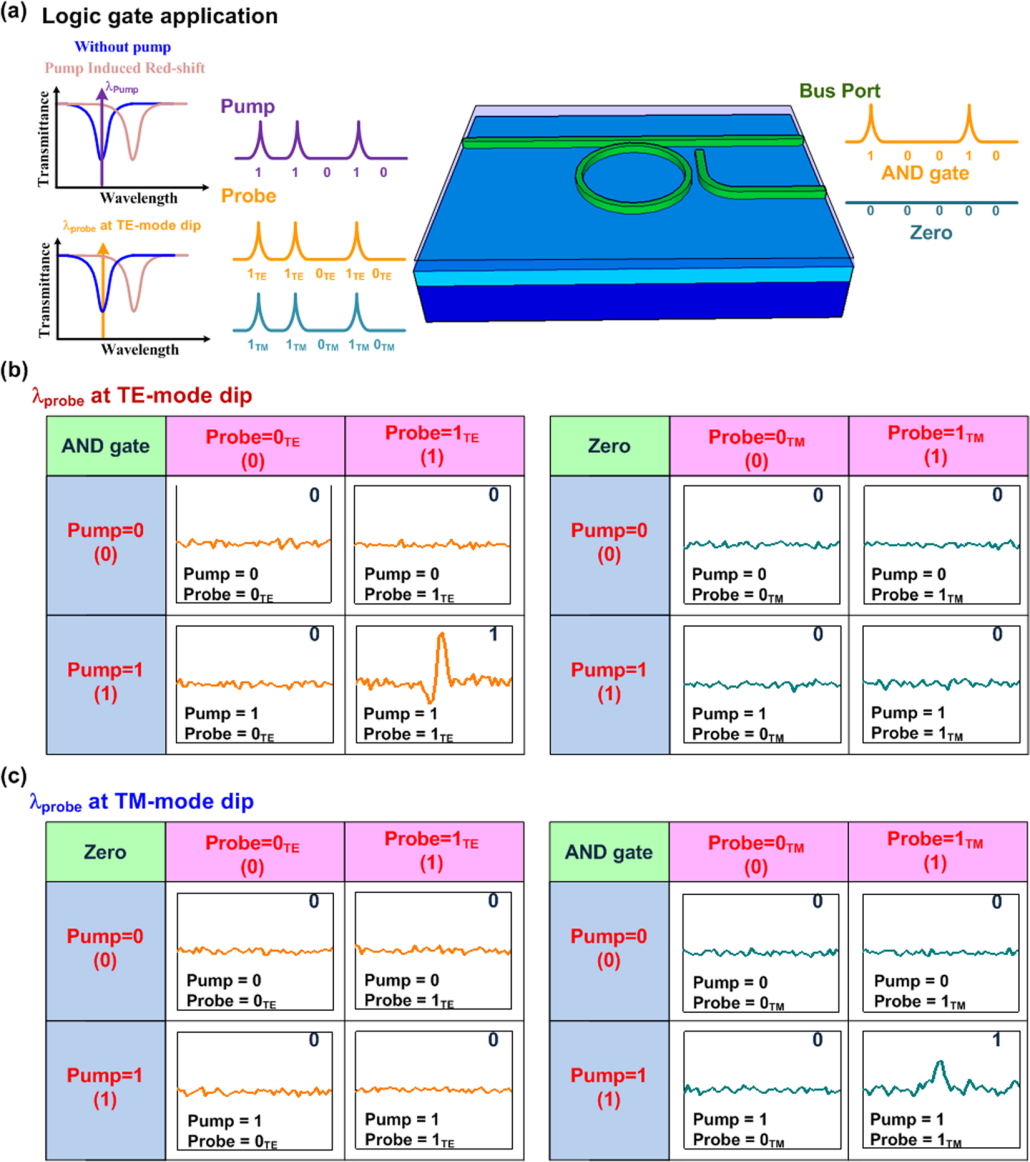
All-optical AND gate application of the C-C bond enriched SiC based add-drop micro-ring. (**a**) The schematic diagram shows C-C bond enriched SiC based micro-ring for logic gate application. (**b**,**c**) The experimental results of all-logic gate application under different cases (pump = 0 or 1, probe = 0_TE/TM_ or 1_TE/TM_)are shown when the probe wavelength is set at TE-mode or TM-mode notched dip.

To perform the all-optical AND gate with the C-C bond enriched SiC based add-drop micro-ring, an intense one-bit pulse with a specific polarization is launched into the add-drop micro-ring, and the pump wavelength is set at the TE-mode notched dip. Because of the TE/TM dependency of the add-drop micro-ring, the probe polarization must be controlled at purely TE or TM polarization (1_TE_ or 1_TM_) by using an in-line polarizer and a polarization controller, as shown in Fig. 8(a). On the other hand, the probe light can also be filtered out by tuning its polarization, which could be denoted as 0_TE_ or 0_TM_. For convenience, the TE and TM dependent truth tables of the add-drop micro-ring are listed in Tables 3 and 4.

**Table 3:** Truth table of all-optical logic gate for probe wavelength at TE-mode dip.

Pump	Probe (λ at TE dip)	Output: AND	Output
0	0_TE/TM_ (TE/TM off)	0_TE_	0_TM_
0	1_TE/TM_ (TE/TM on)	0_TE_	0_TM_
1	0_TE/TM_ (TE/TM off)	0_TE_	0_TM_
1	1_TE/TM_ (TE/TM on)	1_TE_	0_TM_

**Table 4:** Truth table of all-optical logic gate for probe wavelength at TM-mode dip.

Pump	Probe (λ at TM dip)	Output:	Output AND
0	0_TE/TM_ (TE/TM off)	0_TE_	0_TM_
0	1_TE/TM_ (TE/TM on)	0_TE_	0_TM_
1	0_TE/TM_ (TE/TM off)	0_TE_	0_TM_
1	1_TE/TM_ (TE/TM on)	0_TE_	1_TM_

If the probe wavelength is set at the TE-mode notched dip, only the TE-polarized probe can sense the spectral red-shift induced by pump pulse set at ON-state, i.e. pump = 1 and probe = 1_TE_. When probe or pump is set at OFF-state (pump = 0 or probe = 0_TE_), respectively, the output would transfer to OFF-state to implement the AND gate, as shown in Table 3. Simultaneously, when probe is set at TM-polarization or the probe is at OFF-state, the output also transfers to OFF-state. When the probe wavelength is controlled at the TM-mode related dip, the corresponding truth table of all-optical logic gate is listed in Table 4, which is similar to that of the TE case. As a result, the TE-mode and TM-mode dependent results of all-optical logic gate are shown in Fig. 8(b,c). As expected, only if the pumping pulse is set at ON-state with selected wavelength and the polarized probe matches to the TE (or TM) notch dependent wavelength, the output bit can transfer to ON-state.

## Discussion

The TE/TM polarization discriminated data decoder and optical logic gate made by PECVD grown C-C bond enriched SiC film based add-drop micro-ring waveguide with built-in wavelength conversion and format inversion functionalities are demonstrated for the first time. The extraordinary synthesis with a [CH_4_]/([CH_4_] + [SiH_4_]) fluence ratio of 0.96 results in the SiC film with 51% excessive carbon composition, which enriches graphene-like C-C bonds in such a nonstoichiometric C-C bond enriched SiC film to enhance its optical nonlinearity. The C-C bond enriched SiC enhances its nonlinear refractive index up to 2.44 × 10^−12^ cm^2^/W, which is two orders of magnitude larger than that of bulk silicon. With the asymmetric waveguide configuration, the C-C bond enriched SiC add-drop micro-ring exhibits different group indices for TE and TM modes (*N*_*g*_^*TE*^ = 1.705 and *N*_*g*_^*TM*^ = 1.645), thus providing two distinct groups of transmission spectral combs for enabling the TE/TM polarized data decoding. The add-drop micro-ring reveals more polarization sensitive behavior on TE/TM polarized decoding than the bus waveguide which shows inherently different propagation losses for TE and TM modes, and its TE/TM polarized decoding performances at different data rates of 4, 6 and 12 Gbit/s are comparable with those obtained from a commercial 25-dB in-line fiber polarizer. Such an add-drop micro-ring provides dull output ports with a controlled Q-factor at 8 × 10^3^ and a corresponding MER of 8.76 dB in this case. In principle, there is a trade-off between the Q-factor and the MER. As the Q-factor controlled at slightly low value will lead to the shallow notch with the smaller MER, the on-off extinction ratio of the modulated output from the add-drop micro-ring becomes small. In contrast, if the high Q-factor resonator which acts as narrow notch filter is used in this experiment, which is inevitable to spectrally filter out the first and high-order sidebands of the PRZ-OOK data embedded on the pump pulse carrier, and thus limit the bandwidth. Therefore, a properly controlled Q-factor below 10^4^ is mandatory to perform the TE/TM polarized decoding of data-stream at a data rate of 12 Gbit/s; however, the complementary transmission spectra at bus and micro-ring waveguide ports simultaneously show wavelength conversion, and format inversion functionalities at data switching speed of 1.2, 6 and 12 Gbit/s. The modulated output at 1.2 and 6 Gbit/s can pass the criterion set for CMOS to guarantee the operation at bandwidth of 10 Gbit/s for the add-drop micro-ring based. The currently achievable bandwidth of such a wavelength-converted format inversion is merely 10 Gbit/s, because the notch-shaped transmittance spectrum of add-drop micro-ring provides an allowable bandwidth for operating such function. Beyond bandwidth would gradually distort the PRZ-OOK data format after passing through the add-drop micro-ring. Due to the TE/TM polarization dependency of the add-drop micro-ring, the all-optical AND gating can also be demonstrated. For the proposed AND logic, the output can transfer to ON-state only if the polarized pumping pulse and probe is set at ON-state at properly selected wavelength which matches with the corresponding TE/TM notch. This work successfully demonstrates an add-drop micro-ring for implementing 12-Gbit/s TE/TM polarized decoding, 10-Gbit/s dull-port wavelength-converted format inversion and all-optical logic gating functions. As urgent need on high-speed data processing, the on-chip multi-function operation of such an add-drop micro-ring would attract more attention for versatile applications in photonic integrated circuits.

## Methods

### Fabrication of SiC Based Add-drop Micro-ring

The SiC film was deposited on a 3-µm thermal oxide wafer by using a plasma enhanced chemical vapor deposition (PECVD)^43^. During synthesis, a gaseous mixture of 200-sccm methane (CH_4_) and 105-sccm Ar-diluted silane (8% SiH_4_ + 92% Ar) was sent into the chamber. The chamber pressure and substrate temperature were kept at 40 Pa and 550 °C, respectively. A 350-nm SiC film was deposited with a RF plasma power of 120 W and deposition time of 12 min. The waveguide structure was patterned by using e-beam lithography afterwards. Before reactive-ion etching (RIE), Cr hard mask was deposited on the patterned structure by using e-gun. An optimized gaseous mixture of CHF_3_ and O_2_ was applied during the RIE process. Furthermore, a 2-μm SiO_2_ layer was deposited on the waveguide structure, which finally delivers the SiC based add-drop micro-ring.

### Analytic setup of TE/TM polarized decoder, all-optical Kerr switching and all-optical logic in the C-C bond enriched SiC based add-drop micro-ring

For the following experiments, the experimental setup is mainly divided into three parts. The first orange block servers as a TE/TM polarized data decoded probe source, which consists of a tunable laser source (TL, Agilent, 8164 A), a polarization controller, a phase modulator and an arbitrary waveform generator (AWG, Tektronix, 7122B) delivered electrical data. A linearly polarized light with 45° incident angle was modulated by the phase modulator to deliver the optical TE/TM polarized data^44^. The optical TE/TM polarized data was then amplified by an EDFA. In addition, the second purple block shows the experimental setup of pulsed pumping source, and it consists of a tunable laser (TL, HP, 8168 F) source, a 40-GHz Mach-Zehnder modulator (MZM, JDSU, 10024180) and an AWG delivered electrical pulse-train with a pulsewidth of 83 ps. By null-biasing the MZM, the optical pumping pulse was obtained. To achieve sufficiently high peak power with minimal intensity noise for the pumping pulse, it was interactively amplified and filtered by using two-stage erbium-doped fiber amplifiers (EDFAs, JDSU, OAB1552 + 20FA6 & SDO, EFAH1B111NC02) and optical band-pass filters (OBPFs, SANTEC, OTF-910 & JDS, TB1500B). Finally, the blue block is composed of a tunable laser (TL, Agilent, 8164 A) and an EDFA, which acts as a continuous-wave (CW) probe source. For implementing the TE/TM switchable TE/TM polarized decoder, the optical TE/TM polarized data was sent into the polarization-selective add-drop micro-ring. Besides, for cross-wavelength data conversion and inversion with Kerr effect, both the pumping pulse and the CW probe are launched into the to the SiC add-drop micro-ring by using a 50/50 coupler. Moreover, an additional OBPF was employed to filter out the pumping pulse. For all-optical logic application, the CW probe with TE/TM polarization was selected by using an in-line polarizer. Finally, the results of bus- and ring-port outputs are received by using a photo diode (PD, Nortel, pp-10G) and analyzed by using a digital sampling oscilloscope (DSO, Agilent, 86100 A + 86109B).
